# Effect of Deep Margin Elevation on the Fracture Resistance of Maxillary Molar Zirconia Crowns

**DOI:** 10.3390/bioengineering13091057

**Published:** 2026-09-11

**Authors:** Silvia Rojas-Rueda, Domingo Santos Pantaleon, Brian R. Morrow, Franklin Garcia-Godoy, Carlos A. Jurado, Damian Lee, Abdulrahman Alshabib, Mark A. Antal

**Affiliations:** 1 School of Dental Medicine, Ponce Health Sciences University, Ponce 00716, Puerto Rico; 2 Health Research Institute, Faculty Health Sciences, Autonomous of University of Santo Domingo, Santo Domingo 10105, Dominican Republic; 3 Institute for Dental Education and Research, San Francisco de Macorís 31000, Dominican Republic; 4 Department of Bioscience Research, College of Dentistry, The University of Tennessee Health Science Center, Memphis, TN 38163, USA; 5 Department of Prosthodontics, School of Dental Medicine, Tufts University, Boston, MA 02111, USA; 6 Department of Restorative Dental Science, College of Dentistry, King Saud University, Riyadh 11545, Saudi Arabia; 7 Department of Operative and Esthetic Dentistry, Faculty of Dentistry, University of Szeged, 6720 Szeged, Hungary

**Keywords:** crowns, deep margin elevation, fracture resistance, zirconia

## Abstract

Background: The purpose of this in vitro study was to evaluate the fracture resistance of maxillary molar zirconia crowns cemented with deep margins relocated with resin composite. Methods: A maxillary right first-molar typodont tooth was prepared for a complete-coverage zirconia crown and modified to have: (1-DME) a 3 mm deep and 3 mm wide box on the mesial margin; (2-DME) 3 mm deep and 3 mm wide boxes on both the mesial and distal margins; and (N-DME) no box modification. Before the first modification, 45 zirconia (4 mol% yttria) crowns were fabricated. The die was scanned before each modification to fabricate the corresponding 3D-printed dies (*n* = 15 per group). The DME boxes were restored with universal resin composite, and the crowns were then cemented to their respective dies using a resin luting cement. Then artificial aging was performed by thermocycling for 10,000 cycles between 5 °C and 55 °C with a dwell time of 30 s. The cemented crowns were then loaded to failure in a universal testing machine. Maximum fracture load was analyzed with one-way ANOVA and the post hoc Tukey HSD test (α = 0.05). In addition, fracture patterns were evaluated descriptively with scanning electron microscopy images. Results: The fracture resistance of maxillary molar zirconia crowns differed significantly among the groups. Group 2-DME demonstrated the highest mean fracture resistance (5370 N), followed by Group 1-DME (5083 N). The lowest mean value was recorded for the control group, N-DME (2961 N). Conclusions: Zirconia crowns cemented on teeth with deep margin elevation exhibited higher fracture resistance than crowns cemented without deep margin elevation.

## 1. Introduction

Restorative dentists often encounter deep caries, defective restorations, and fractures extending into the cervical tooth structure. Subgingival interproximal defects, especially those located below the cementoenamel junction (CEJ), are particularly challenging because they complicate visualization, isolation, bonding, finishing, and maintenance of the restorative margin [1,2]. Indirect restorations are commonly used for extensively damaged posterior teeth because they allow improved control of proximal contours, occlusal anatomy, contacts, and restoration morphology, while also permitting the use of ceramic materials with favorable mechanical properties [3]. Unlike resin composites, ceramics are not affected by polymerization shrinkage, which may help preserve restoration geometry, marginal adaptation, and proximal contacts [4,5].

Zirconia can be classified according to its yttria (Y_2_O_3_) content. The most commonly used types are 4Y and 5Y zirconia, while 3Y zirconia is less commonly used in current restorative applications. 4Y zirconia contains approximately 4 mol% yttria, which corresponds to about 7% to 8% by weight, whereas 5Y zirconia contains approximately 5 mol% yttria, corresponding to about 8% to 9.5% by weight. In general, 4Y zirconia provides greater fracture resistance and is commonly used in posterior regions of the mouth, where restorations are subjected to higher occlusal forces. In contrast, 5Y zirconia has lower fracture resistance but greater translucency, resulting in improved optical properties. For this reason, 5Y zirconia is more commonly used in the anterior region, where esthetics are a primary consideration [6,7,8,9].

The clinical success of zirconia restorations also depends on the quality and location of the margins, the adhesive interface, and the supporting tooth structure. Subgingival margins are difficult to manage because of limited access, compromised isolation, and contamination by saliva, crevicular fluid, or blood. These factors may reduce adhesive performance and marginal integrity and increase the risk of microleakage, secondary caries, and restorative failure [10,11]. Deep margins may also complicate conventional impressions and digital scanning when the finish line is obscured.

When a restorative margin is located deep interproximally, clinicians must decide whether to restore it in its original position or relocate it to a more accessible level. Orthodontic extrusion and surgical procedures such as crown lengthening or gingivectomy can improve access to sound tooth structure, but they may increase treatment time, cost, invasiveness, and postoperative delay. Crown lengthening may also alter gingival and bone levels, reduce the crown-to-root ratio, increase root exposure, and affect periodontal architecture [12,13,14]. These limitations have increased interest in more conservative alternatives.

Deep margin elevation (DME), also called proximal box elevation or cervical margin relocation, is a nonsurgical technique that uses an adhesive restorative material, typically resin composite, to reposition a deep subgingival margin to a more coronal and accessible level. DME may improve visualization, isolation, matrix adaptation, finishing, and polishing. DME can also facilitate conventional or digital impressions and improve access during adhesive cementation and excess cement removal. By reducing the need for periodontal surgery and preserving gingival and osseous architecture, DME may be used before indirect partial-coverage restorations and full-coverage crowns [15]. Successful clinical applications of DME before indirect restorations have been reported in the literature [16,17,18].

Despite these potential advantages, the introduction of a resin-composite layer beneath an indirect restoration creates an additional restorative interface and changes the structural configuration of the supporting tooth. The elevated area may be subjected to polymerization stresses, occlusal loading, hydrolytic degradation, and fatigue at the tooth–composite and composite–cement interfaces. The extent of the DME restoration may also be relevant. Elevation of a single proximal surface preserves more native tooth structure than elevation of both proximal surfaces, whereas two-surface DME may result in a greater volume of resin composite and a more extensive replacement of cervical tooth structure. These differences could influence stress distribution, crown support, deformation of the restored tooth, and the location or severity of fracture under loading.

This issue may be particularly relevant for maxillary molars because they are exposed to substantial occlusal forces and possess complex anatomical features, including multiple cusps and roots. Although zirconia crowns demonstrate favorable mechanical properties, their fracture behavior may be affected by the geometry and mechanical characteristics of the underlying foundation. Consequently, it is important to determine whether the presence and extent of DME influence the fracture resistance of zirconia crowns placed on molar teeth. However, limited information is currently available regarding the effect of one-surface or two-surface DME on the fracture resistance of full-coverage zirconia molar crowns. Therefore, the aim of this in vitro study was to evaluate the fracture resistance of maxillary first-molar zirconia crowns cemented on teeth with one or two proximal surfaces restored using DME with resin composite and to compare them with zirconia crowns cemented on teeth without margin elevation. The first null hypothesis was that there would be no significant difference in fracture resistance among zirconia crowns cemented on teeth with one-surface DME, crowns cemented on teeth with two-surface DME, and crowns cemented on teeth without margin elevation. The second null hypothesis was that there would be no significant difference in mean fracture resistance between crowns with one-surface DME and those with two-surface DME.

## 2. Materials and Methods

**Figure 1:**
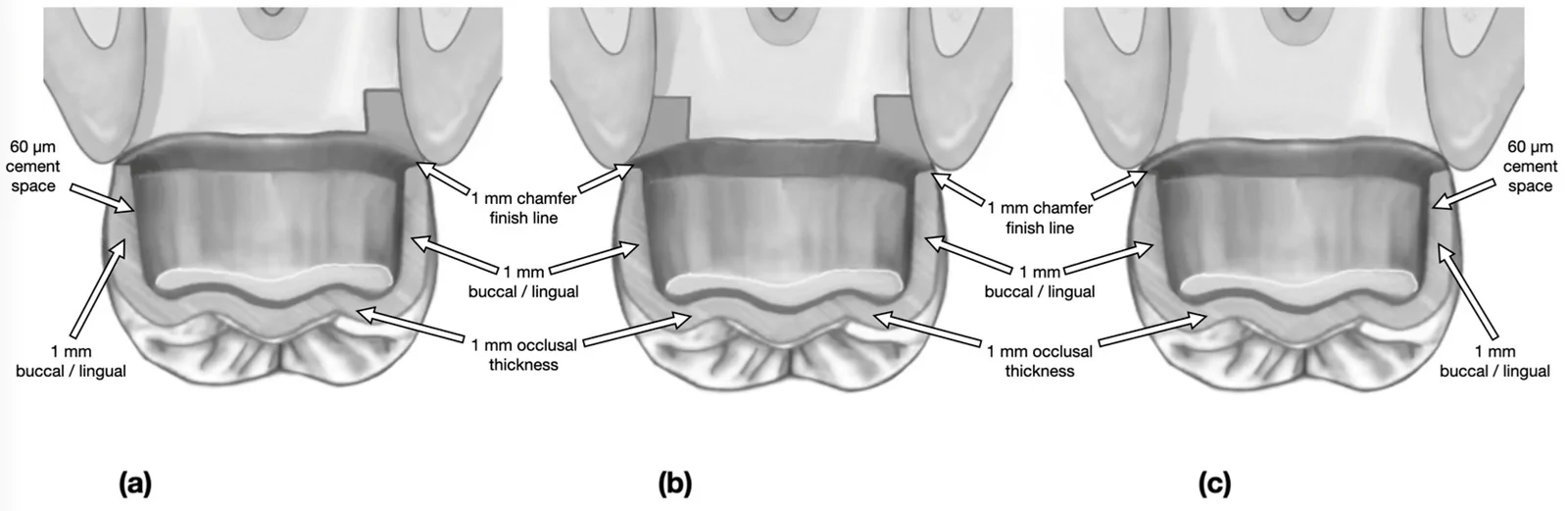
Schematic drawing of the maxillary zirconia crowns cemented with and without deep margin elevation (DME) areas. (**a**) Crowns cemented over mesial margin with DME, (**b**) crowns cemented over mesial and distal margins with DME; (**c**) crowns cemented without DME.

A dental model of an upper right first molar (1560 Series; Columbia Dentoform Inc., Lancaster, PA, USA) was shaped to simulate a tooth prepared to receive a complete ceramic crown. The preparation included 1.0 mm of occlusal reduction, 1.0 mm of buccal and lingual axial reduction, and a 1.0 mm chamfer finish line. The prepared tooth was scanned with a chairside scanner (Primescan; Dentsply Sirona, Charlotte, NC, USA), and the crown was digitally designed (CEREC Software 5.3; Dentsply Sirona, Charlotte, NC, USA) with 60 μm of cement space, 1.0 mm occlusal thickness, 1.0 mm buccal lingual thickness, and a 1.0 mm chamfer finish line, following the occlusal anatomy recommended by the software. The tooth was then modified by creating a 3 mm wide × 3 mm deep box on the mesial margin and rescanned. Finally, a second 3 mm wide × 3 mm deep box was created on the distal margin, and the tooth was scanned again (Figure 1).

Using the first scan file, forty-five (45) crowns (*n* = 15/group) were milled from 4Y zirconia (HT; Katana, Kuraray Noritake Dental Inc, Tokyo, Japan) using a milling machine (DWX-52; Roland DGA). The three die scans were digitally modified (DentalCAD 3.1 Rijeka; exocad, Darmstadt, Germany) to create a 5 mm cylindrical shape with a flat end below the gingival margin of the crown preparation, providing a stable geometry for subsequent testing. Each die from the three scans was printed in resin (Gray Resin; Formlabs, Somerville, MA, USA) using a 3D printer (FormLab 2; Formlabs). Each die was carefully inspected by an experienced prosthodontist to ensure that no defects or imperfections were introduced during the printing procedure. Then, the printed dies were washed in isopropyl alcohol for 20 min (Form Wash; Formlabs, Somerville, MA, USA) and then post-cured with a wavelength of 405 nm light in a UV curing unit (Form Cure; Formlabs) using standard AC electrical power for 30 min according to the manufacturer’s instructions.

The zirconia crowns underwent a 2 h sintering process at a maximum temperature of 1500 °C (2732 °F), as recommended by the manufacturer (HT; Katana, Kuraray Noritake Dental Inc, Tokyo, Japan). The intaglio surfaces of the restorations were then airborne-particle abraded with 50 µm aluminum oxide particles using compressed air at 30 psi for 10 s from a distance of 10 mm at room temperature, followed by cleaning with a paste (Katana Cleaner; Katana Kuraray) for 10 s, rinsing, and drying. The dies with the 3 mm × 3 mm boxes were first cleaned with 34% phosphoric acid (Caulk 34%; Dentsply Sirona), then rinsed and air-dried. A universal adhesive (Prime & Bond Universal; Dentsply Sirona) containing 10MDP (Methacryloyloxydecyl dihydrogen phosphate) PENTA (dipentaerythritol pentaacrylate monophosphate) adhesive water and isopropanol (2-propanol) solvent was then applied, and the boxes were restored using oblique increments of resin composite (TPH Spectra; Dentsply Sirona). It is important to mention that 10-MDP and PENTA are not a single compound; rather, they are distinct chemical components commonly used in modern dental bonding agents. 10-MDP is a functional monomer that can chemically bond to calcium in tooth structure and to metal oxides, whereas PENTA is another functional monomer that contributes to multi-point bonding and cross-linking, thereby enhancing bond strength [19,20,21]. Each increment was light-cured (Elipar 2500; 3M, St Paul, MN, USA) for 20 s, ensuring that the final increment matched the level of the finish margin where the crown would seat. After all increments were placed, a final 20 s cure was applied to the occlusal and proximal surfaces.

**Figure 2:**
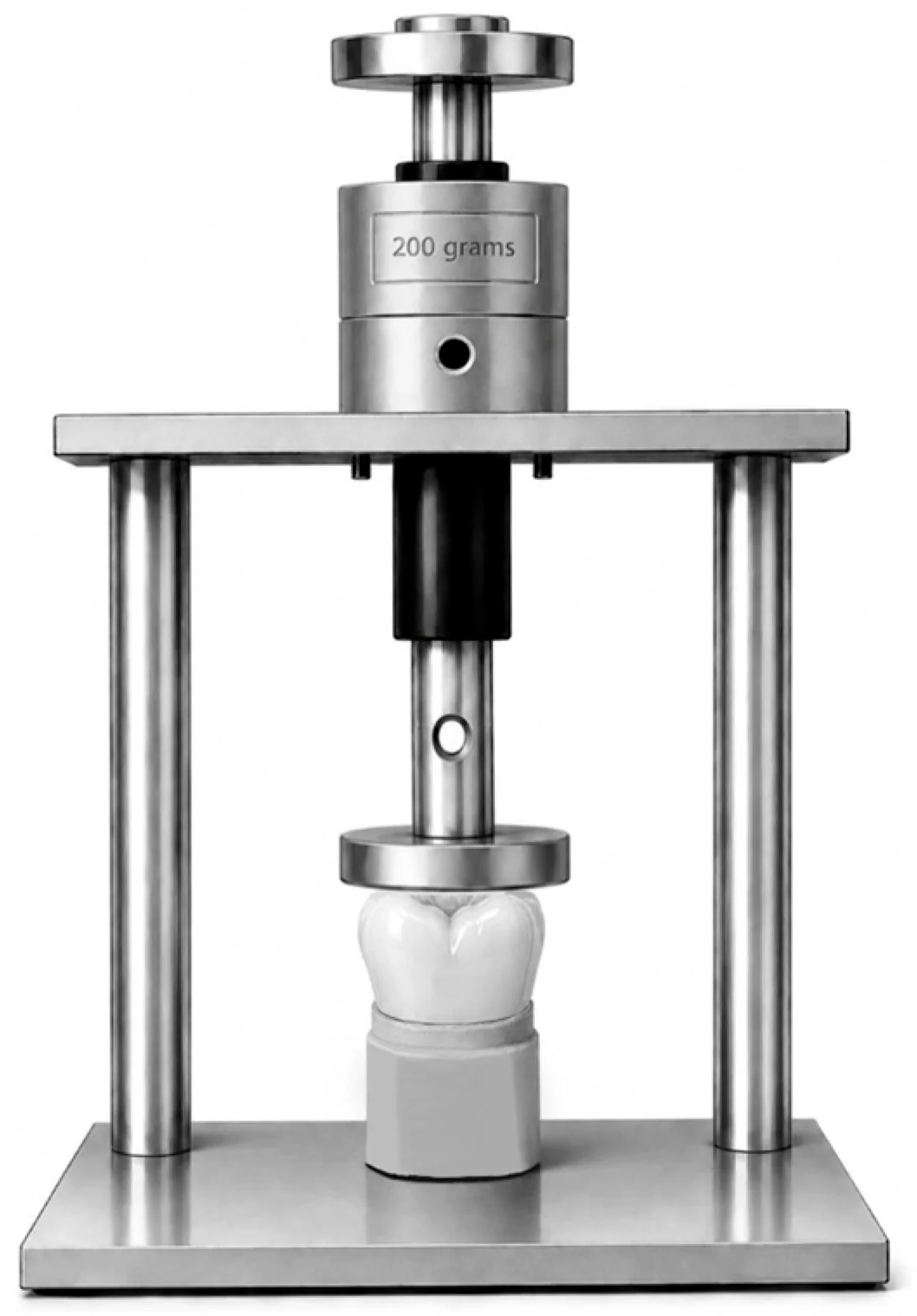
Schematic illustration of a cemented crown under a 200 g load.

A single, experienced prosthodontist evaluated the seating of all crowns on the dies, ensuring that the deep margin elevation (DME) did not interfere with the ideal fit of the restoration. The restorations were then treated with primer (Prime & Bond Universal; Dentsply Sirona) and air-dried and subsequently cemented with a dual-cure adhesive resin cement (Calibra Universal; Dentsply Sirona). Light polymerization was performed with an LED curing light (output 1470 mW/cm^3^, Elipar 2500; 3M) for 20 s each on the occlusal, mesial, distal, buccal, and lingual surfaces. The light output intensity was verified in between each crown cementation with a dental radiometer (Ledex LED Radiometer CM 400, Dentmate Technology Co., New Taipei City, Taiwan). The restorations were then allowed to self-cure for 6 min under a constant 200 g load applied using a calibration weight (200 g Calibration Weight, American Weigh Scales, Cumming, GA, USA) (Figure 2).

**Figure 3:**
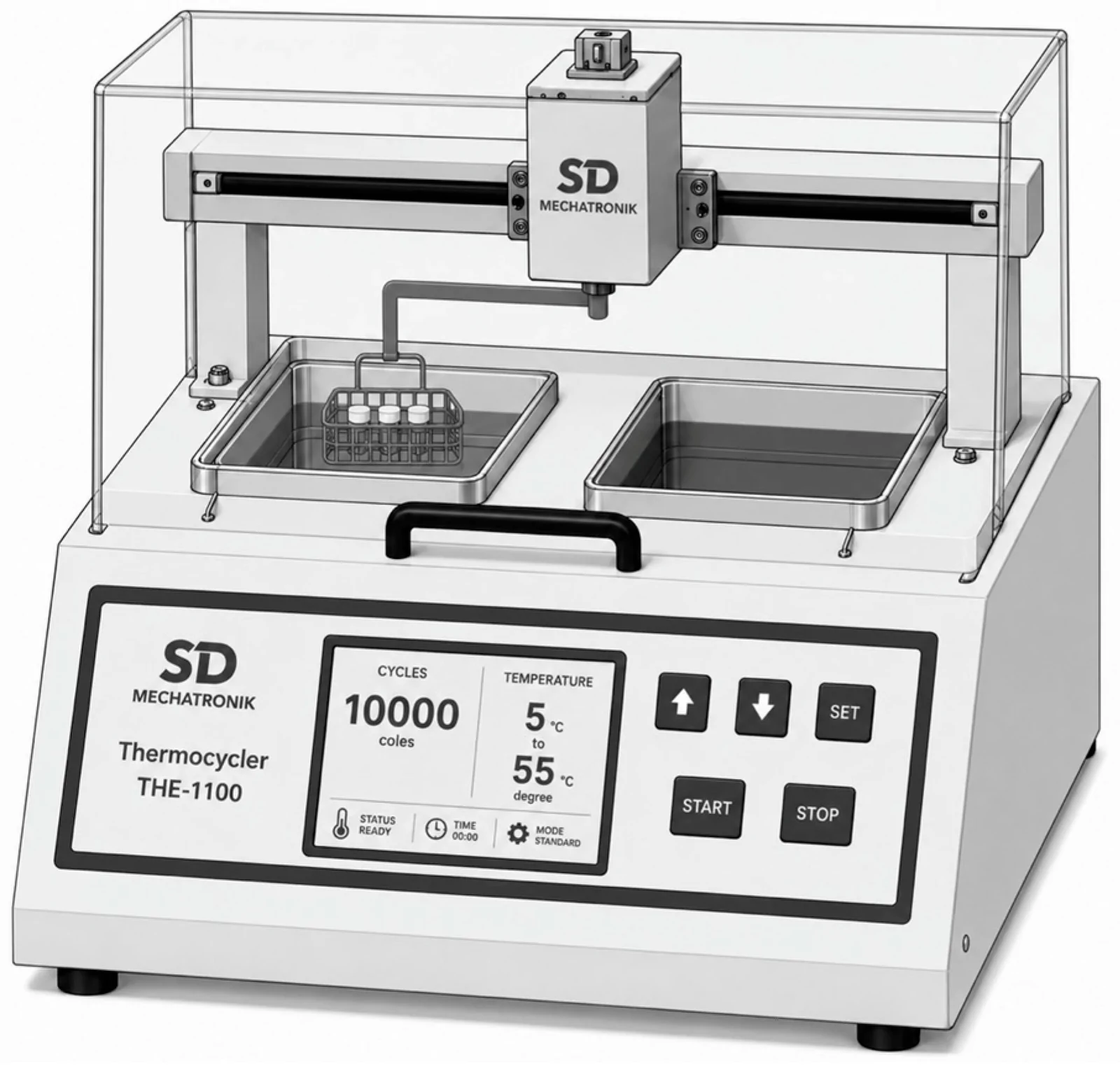
Schematic illustration of the thermocycling process applied to the cemented restorations.

All restorations underwent artificial aging by means of 10,000 thermal cycles in water between 5 °C and 55 °C, with a dwell time of 30 s at each temperature (Thermocycler THE-1100; SD Mechatronik, Westerham, Germany) (Figure 3).

After completion of 10,000 thermal cycles, all specimens were visually examined by a single experienced evaluator using dental loupes at 5× magnification (True Magnification, Designs for Vision, Bohemia, NY, USA) for evidence of crown debonding or fracture. No debonding or visible damage was observed in any of the restorations.

Following aging, the specimens were mounted in a metal base jig to prevent movement during testing. A vertical load was applied to the center of the occlusal surface of each crown at a crosshead speed of 1.0 mm/min until failure using a universal testing machine (4411 Tensile Strength Tester; Instron, Norwood, MA, USA). A tapered, cone-shaped stainless-steel indenter with a 2.00 mm tip was used, and a 1 mm thick rubber sheet was placed between the loading tip and the occlusal surface to simulate food-bolus distribution. The load at which complete fracture occurred was recorded in newtons (N).

**Figure 4:**
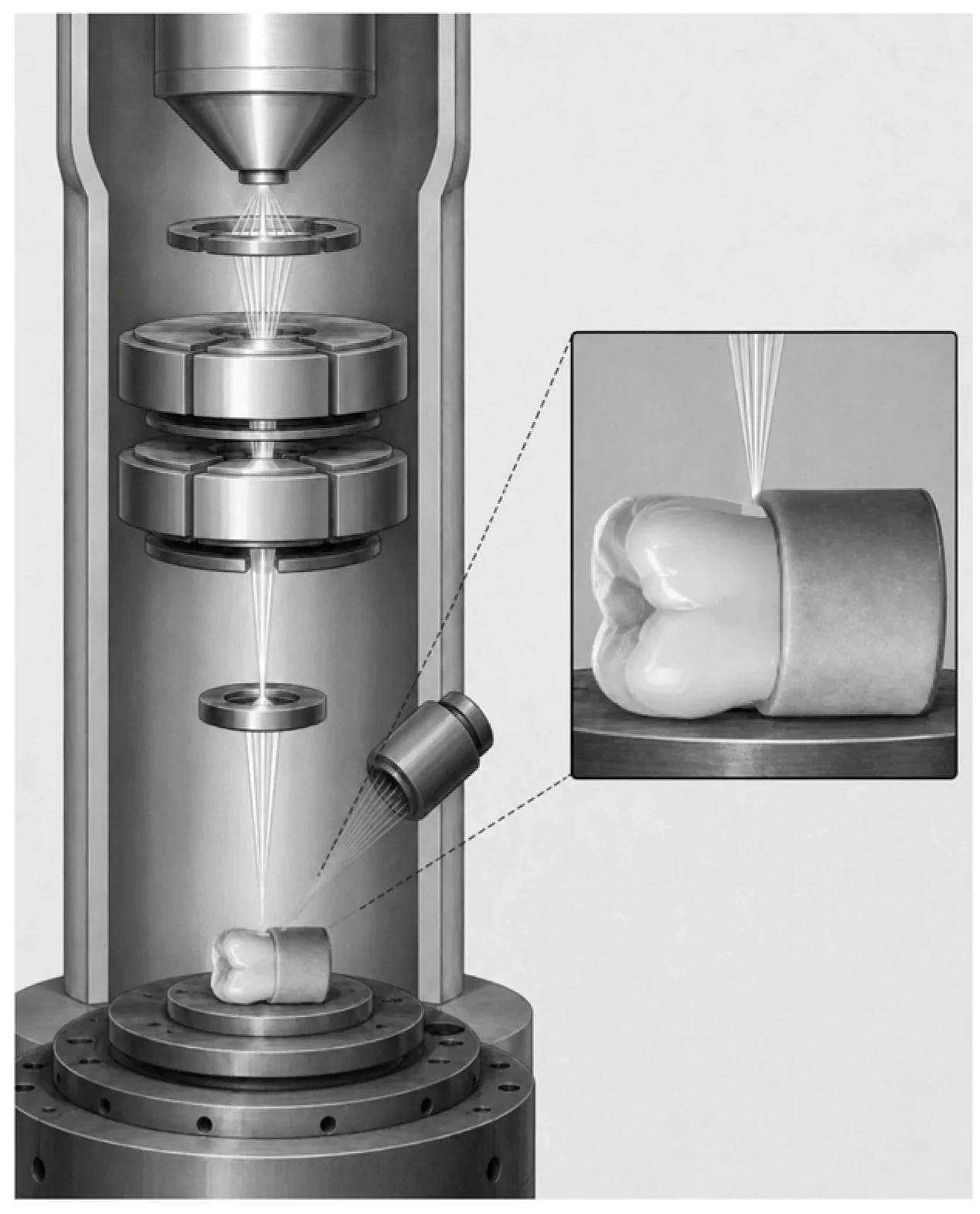
Schematic illustration of the surface evaluated with the scanning electron microscope.

The sample size of 15 specimens per group used in the present study is consistent with previous studies evaluating the fracture resistance of dental restorations, which have typically included 10 to 15 specimens per group [22,23,24]. Data were analyzed using one-way ANOVA and Tukey’s HSD post hoc test (α = 0.05) with SPSS software 32.0 (IBM; Armonk, NY, USA). All fractured specimens were visually assessed by an experienced dental specialist to identify any specific fracture patterns, and one representative specimen from each group was selected. The selected samples received a thin coat of gold using a sputter coater (Quick Coater SC-701; Sanyu Electron, Tokyo, Japan) to ensure electrical conductivity and then scanning electron microscopy (SEM) images were obtained with an accelerating voltage of 15 kV (Figure 4).

## 3. Results

Table 1 presents the fracture resistance of zirconia crowns on maxillary first molars cemented over two-surface DME, one-surface DME, or without DME. The fracture load was significantly affected by the presence of DME. Crowns cemented over two-surface DME showed the highest fracture load (5370 N), followed by crowns cemented over one-surface DME (5083 N). The lowest fracture load was observed in crowns cemented without DME (2961 N). One-way ANOVA revealed a statistically significant difference among groups (F(2,42) = 343.99, *p* < 0.001, η^2^ = 0.94). Tukey post hoc analysis identified statistically significant differences between all groups. The 2-DME group exhibited significantly greater fracture resistance than the 1-DME group (mean difference = 287.80 N; 95% CI, 44.04 to 531.56 N; adjusted *p* = 0.017). The 1-DME and 2-DME groups also exhibited significantly greater fracture resistance than the N-DME group, with mean differences of 2121.53 N (95% CI, 1877.77 to 2365.29 N; adjusted *p* < 0.001) and 2409.33 N (95% CI, 2165.57 to 2653.09 N; adjusted *p* < 0.001), respectively.

**Figure 5:**
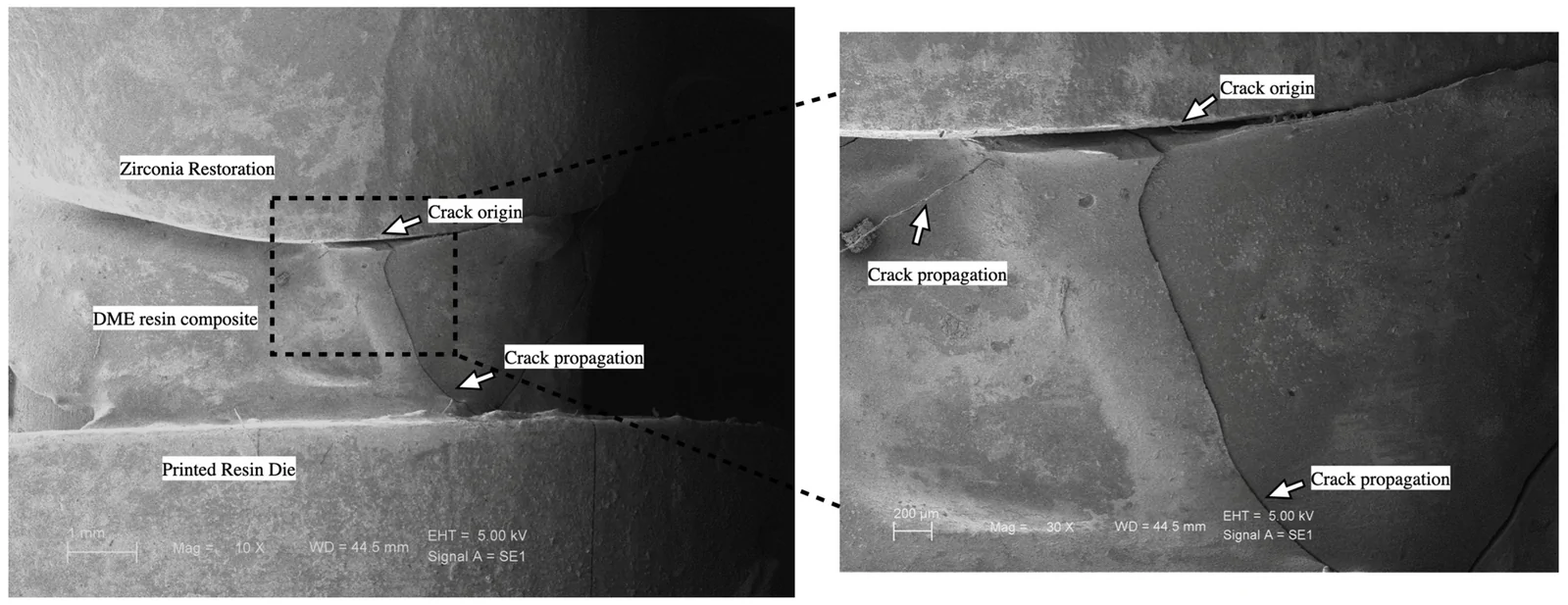
Representative scanning electron microscopy (SEM) images of a fractured crown with mesial DME with 10× and 30× magnification. The figures display the zirconia restoration, DME resin composite, printed resin die, crack origin and crack propagation.

**Figure 6:**
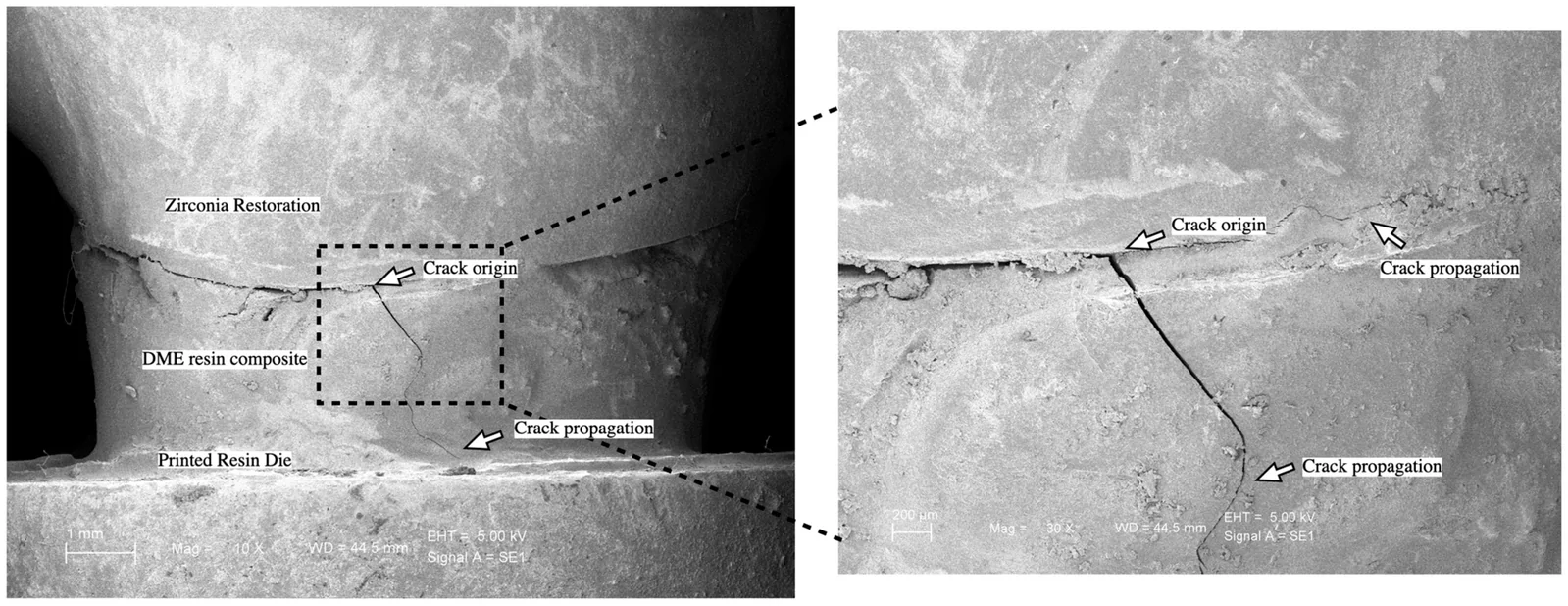
Representative scanning electron microscopy (SEM) images of a fractured crown with mesial and distal DME with 10× and 30× magnification. The figures display the zirconia restoration, DME resin composite, printed resin die, crack origin and crack propagation.

**Figure 7:**
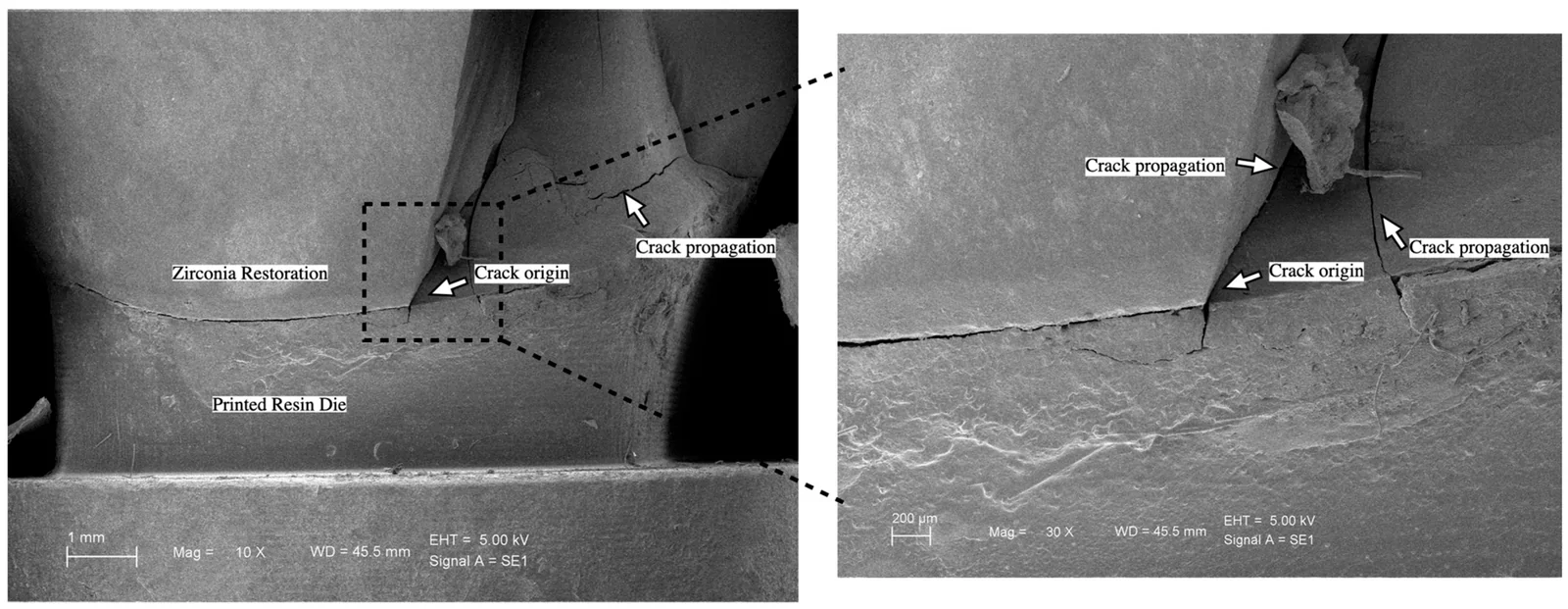
Representative scanning electron microscopy (SEM) images of a fractured crown with N-DME with 10× and 30× magnification. The figures display the zirconia restoration, N-DME, printed resin die, crack origin and crack propagation.

Representative scanning electron microscope (SEM) images of the fractured specimens at 10× and 30× magnification are shown in Figure 5, Figure 6 and Figure 7.

## 4. Discussion

The present study compared the fracture resistance of maxillary molar zirconia crowns cemented on preparations with one-surface DME (1-DME), two-surface DME (2-DME), or no DME (N-DME) after thermocycling. Significant differences were found among all of the groups (*p* < 0.001), leading to rejection of the first null hypothesis. The 1-DME and 2-DME groups exhibited higher mean fracture resistance values (5083 N and 5370 N, respectively) than the N-DME group (2961 N), and a significant difference was found between the 1-DME and 2-DME groups. Therefore, the second null hypothesis was also rejected. Although fracture resistance values were high in all groups, DME did not compromise the fracture resistance of zirconia crowns under the conditions evaluated and was associated with greater fracture resistance than the absence of DME. These findings indicate that, within the 3D-printed resin-die model used in this study, replacing a portion of the cervical resin-die structure with resin composite for simulated margin relocation did not create a mechanically weaker foundation for the zirconia crowns tested.

Deep proximal margins were simulated using standardized mesial and distal box preparations measuring 3 mm in width and 3 mm in depth and were restored with resin composite to the level of the cervical finish line before crown cementation. These dimensions are consistent with clinical reports describing relocation of margins located approximately 2 to 3 mm subgingivally [25,26] and are comparable with the 3 to 3.5 mm height of some prefabricated interproximal matrix bands [27]. Standardizing the dimensions and geometry of the DME preparations also minimized variability in resin composite volume, polymerization shrinkage, adaptation, interfacial stress, and mechanical support, allowing a more direct assessment of the influence of one- and two-surface DME on fracture resistance.

The available literature contains limited information regarding the fracture resistance of posterior zirconia crowns cemented on preparations with DME. Nevertheless, the findings of the present in vitro study are consistent with those of previous investigations involving other indirect restoration designs and ceramic materials placed over elevated margins. One study evaluated the effects of DME and preparation design on the fracture strength of indirectly restored molars. Sixty extracted teeth were restored with CAD-CAM lithium disilicate inlays or onlays, either with or without DME. The restorations were subjected to thermomechanical aging and subsequently loaded until fracture. Higher fracture-load values were reported for both restoration designs when DME was used. The mean values were 1194 N for inlays without DME, 1393 N for inlays with DME, 1776 N for onlays without DME, and 1986 N for onlays with DME. The authors concluded that restorations both with and without DME demonstrated fracture resistance values that were likely to withstand clinical functional loading [28].

Another recent in vitro study evaluated the effect of DME on the fracture resistance of premolars restored with lithium disilicate onlays. Five experimental groups were investigated: onlays without DME; onlays with a 2 mm DME made from bulk-fill resin composite; onlays with a 2 mm DME made from short-fiber-reinforced flowable resin composite; onlays with a 3 mm DME made from bulk-fill resin composite; and onlays with a 3 mm DME made from short-fiber-reinforced flowable resin composite. After cementation, the restorations underwent artificial aging through thermocycling and were subsequently loaded until fracture. The group without DME demonstrated the lowest mean fracture resistance value, at 857 N. The DME groups demonstrated higher values: 914 N for the 2 mm bulk-fill DME group, 935 N for the 3 mm bulk-fill DME group, 1145 N for the 2 mm short-fiber-reinforced DME group, and 1182 N for the 3 mm short-fiber-reinforced DME group. The authors concluded that margin elevation with resin-based materials resulted in higher fracture resistance than restorations without margin elevation [29].

Although previous studies evaluated lithium disilicate partial-coverage restorations rather than zirconia full-coverage crowns, their findings suggest that a resin-composite foundation beneath an indirect restoration does not necessarily reduce fracture resistance. Direct comparisons are limited by differences in ceramic type, restoration design, tooth type, luting protocol, and loading conditions. Nevertheless, similar or higher fracture resistance reported for DME groups supports resin composite as a potentially effective supporting substrate.

Clinical evidence also supports the use of DME. In a study of 197 indirect posterior restorations in 120 patients followed for up to 12 years, only eight failures occurred, corresponding to a 95.9% survival rate [30]. Similarly, a recent systematic review of eight clinical studies with follow-up periods ranging from 3 months to 12 years reported survival rates of 94% to 100% for restorations placed with DME [31]. The review concluded that favorable restorative and periodontal outcomes can be achieved when DME is performed with appropriate isolation, adhesive technique, finishing, polishing, and maintenance [22]. Accordingly, clinical success depends not only on material properties but also on proper case selection, moisture control, matrix adaptation, polymerization, and finishing of the elevated margin.

One possible explanation for the higher fracture resistance observed in the DME group is the mechanical behavior of the resin composite used to replace the cervical portion of the preparation. Dentin has a reported compressive strength of approximately 250–350 MPa [32,33], whereas the manufacturer reports a compressive strength of 400 MPa for the resin composite used in this study. These values suggest that the resin-composite foundation may have provided compressive support comparable to that of dentin, although this relationship should be interpreted cautiously.

Flexural behavior may also have influenced the results observed in this study. The maximum stress reported for dental enamel, which can be considered in relation to its flexural strength, is approximately 62 MPa [34]. By comparison, the manufacturer reports a flexural strength of 135 MPa for the resin composite used in the present investigation [35]. Although direct comparisons between values obtained from different testing methods and sources should be interpreted with caution, these reported properties suggest that the composite may have been sufficiently resistant to deformation and crack development during loading. Accordingly, the resin composite may have provided additional support in the elevated cervical area and contributed to a more favorable stress distribution beneath the zirconia crown.

The resin composite used for DME may also have functioned as an intermediate stress-modulating layer between the relatively rigid zirconia crown and the supporting die. Because resin-based materials generally have a lower elastic modulus than zirconia, they may undergo a limited degree of elastic deformation under loading. This behavior could potentially decrease stress concentration at the crown margin and redistribute part of the applied load through the underlying composite. Such a mechanism may help explain why the DME groups tolerated higher loads before catastrophic failure. However, because stress distribution and interfacial strain were not directly measured in the present study, this interpretation remains theoretical and should be further investigated using finite element analysis, strain-gauge measurements, or other biomechanical approaches.

A statistically significant difference was observed between the 1-DME and 2-DME groups, indicating that, within the 3D-printed resin-die model, increasing the number of elevated proximal surfaces from one to two was associated with a significant increase in the fracture resistance of the zirconia crowns. The two-surface DME configuration involved a greater volume of resin composite and replacement of a larger portion of the cervical 3D-printed resin-die structure. Under the experimental conditions, this configuration resulted in a measurable increase in fracture resistance. However, these findings should not be extrapolated directly to natural teeth, and the effects of more extensive DME configurations, larger box dimensions, or circumferential margin relocation require further investigation.

SEM observations provided additional context for the fracture behavior. In the DME group, crack lines extended through the cervical resin composite, indicating that the composite was involved in stress transfer during failure. In contrast, no visible cracks extended into the supporting die in the N-DME group. These findings should be interpreted cautiously, as crack patterns may also be influenced by material properties, interfacial adhesion, restoration geometry, and local stress concentration. Further fractographic analysis would be needed to identify the origin and direction of crack propagation.

Although three-dimensionally printed resin dies were used instead of natural teeth, the resin had a reported tensile strength of 61 MPa, within the range reported for dentin (44–97.8 MPa) [36,37]. Three-dimensionally printed resin dies were used as standardized experimental substrates in the present study. The use of printed dies allowed consistent control of specimen dimensions, preparation geometry, margin location, and restoration design and reduced variability among specimens. Similar resin-die models have been used in previous fracture resistance investigations [38,39]. Nevertheless, the mechanical behavior of a 3D-printed resin substrate cannot be assumed to reproduce that of natural dental tissues, and the present findings should therefore be interpreted specifically within the resin-die model used in this investigation. In addition, a recent study found no significant difference in the fracture resistance of zirconia crowns cemented on resin dies versus natural teeth [40]. Printed dies also allowed consistent standardization of tooth dimensions, preparation geometry, margin location, and restoration design.

The artificial aging protocol should also be considered when interpreting the findings. Thermocycling is a commonly used artificial aging method in dental research in which restorations are repeatedly exposed to alternating hot and cold water baths. This process simulates thermal stresses that occur in the oral environment and helps evaluate the long-term durability of dental materials and restorations. Thermocycling exposes specimens to repeated temperature changes and may produce expansion and contraction of the different restorative materials, potentially stressing the bonded interfaces. However, thermocycling alone does not fully reproduce the complex oral environment. Intraoral restorations are also exposed to cyclic occlusal forces, lateral loading, pH fluctuations, enzymatic activity, water sorption, and progressive fatigue over time. Therefore, the high fracture resistance values recorded after thermocycling should not be interpreted as direct predictors of long-term clinical survival.

This study has several limitations. First, only one zirconia material was evaluated. Clinicians currently have access to a wide range of zirconia formulations that differ in yttria content, translucency, grain structure, flexural strength, and resistance to crack propagation. Other ceramic materials, including leucite-reinforced ceramics, lithium disilicate, zirconia-reinforced lithium silicate, and hybrid ceramics, may respond differently when supported by DME. Future studies should therefore assess additional restorative materials and compare their mechanical behavior under standardized conditions.

Second, only maxillary first-molar crowns were evaluated. Tooth anatomy, preparation dimensions, crown thickness, and loading direction differ among maxillary molars, mandibular molars, and premolars. These differences may influence stress distribution and fracture resistance. Future investigations should include other posterior teeth to provide a broader understanding of the biomechanical effects of DME throughout the posterior dentition.

Third, only one resin composite and one DME protocol were investigated. The mechanical behavior of an elevated margin may vary according to the type of resin composite, filler content, elastic modulus, polymerization shrinkage, depth of cure, and placement technique. Flowable composites, conventional composites, bulk-fill composites, and fiber-reinforced materials may provide different levels of support. Future studies should compare these materials and evaluate the effects of different DME heights, widths, and configurations.

Fourth, although resin composite is commonly used for DME, glass-ionomer and resin-modified glass-ionomer materials have also been described for cervical margin relocation. These materials differ from resin composites in strength, elastic modulus, adhesion, water sensitivity, and fluoride release. Future studies should evaluate the fracture resistance of zirconia crowns cemented on preparations in which DME is performed with glass-ionomer-based materials.

Additional limitations include the use of a static axial load to failure and the absence of mechanical fatigue loading. A single compressive load does not reproduce the repetitive and multidirectional forces generated during mastication or parafunctional activity. Future investigations should combine thermocycling with cyclic mechanical loading and should apply forces at clinically relevant angles. Evaluation of marginal adaptation, microleakage, bond durability, failure mode, and periodontal response would also provide a more comprehensive assessment of the clinical implications of DME.

Another limitation of the present study is the use of 3D-printed resin dies rather than natural teeth. Although natural dentition may provide a closer approximation of clinical conditions, the use of printed dies can help standardize specimen dimensions and reduce variability among samples. Natural teeth may be affected by dehydration during experimental procedures, and repeated manual preparations can introduce operator-dependent differences. In addition, obtaining a sufficient number of caries-free teeth with comparable size, morphology, and structural characteristics can be difficult. Future studies may benefit from incorporating natural dentition while applying appropriate methods to minimize these potential sources of variability. Furthermore, periodontal ligament simulation was not incorporated into the present study; therefore, future studies may consider including it. Periodontal ligament simulation could provide additional information regarding the distribution and transmission of occlusal forces and their potential effects on the supporting periodontal tissues, which may influence the clinical behavior and long-term success of the restoration. Another limitation is the fractographic analysis, which included only one specimen per group. Future studies should evaluate a greater number of specimens using SEM to provide a more comprehensive visual assessment of crack patterns, including their morphology, distribution, and length.

Within the limitations of this in vitro study and the 3D-printed resin-die model used, simulated relocation of one or two deep proximal margins with resin composite was associated with greater fracture resistance of maxillary molar zirconia crowns compared with crowns cemented on resin-die preparations without DME. A statistically significant difference was also identified between the one-surface and two-surface DME configurations. These findings indicate that resin-composite margin elevation did not compromise the fracture resistance of zirconia crowns within the experimental model. However, the results should not be directly extrapolated to natural teeth, and further laboratory studies using natural dental substrates and clinical investigations are required.

## 5. Conclusions

Within the limitations of this in vitro study, deep margin elevation (DME) with a resin composite did not compromise the fracture resistance of maxillary molar crowns fabricated from 4Y zirconia. Crowns cemented on preparations with one-surface DME and two-surface DME demonstrated significantly higher mean fracture resistance values (5083 N and 5370 N, respectively) than 4Y zirconia crowns cemented on preparations without DME (2961 N). These findings indicate that, under the tested conditions, the presence of DME was associated with greater fracture resistance of 4Y zirconia crowns, and increasing the number of DME surfaces from one to two provided an additional statistically significant effect. These results should be interpreted specifically within the context of the 4Y zirconia material evaluated and should not be directly extrapolated to zirconia with different yttria concentrations or other restorative materials. Furthermore, the findings should be interpreted with caution, as several clinically relevant factors that cannot be fully reproduced in laboratory studies, such as adequate field isolation during resin composite placement, the patient’s occlusal conditions, oral hygiene, periodontal status, and other intraoral variables, may influence the long-term clinical performance of restorations involving DME.

## Figures and Tables

**Table 1:** Mean and standard deviation values of the fracture resistance values obtained for zirconia restorations cemented on dies with mesial DME (1-DME), mesial and distal DME (2-DME), and no DME (N-DME).

Group	Type of Restoration	Mean and Standard Deviation of the Fracture Resistance Values
1-DME	Zirconia crowns cemented on DME on the mesial surface	5083 ± 348 N ^b^
2-DME	Zirconia crowns cemented on DME on the mesial and distal surfaces	5370 ± 249 N ^a^
N-DME	Zirconia crowns cemented without any DME	2961 ± 206 N ^c^

DME, deep margin elevation; N, newtons. Different superscript letters indicate significant difference (*p* < 0.05).

## Data Availability

The original contributions presented in the study are included in the article; further inquiries can be directed to the corresponding authors.
